# Presenilin-1 C779T Mutation Presenting With Rapidly Progressive Dementia and Medial Temporal Lobe MRI Changes

**DOI:** 10.1155/crnm/8251065

**Published:** 2025-11-14

**Authors:** Marco Toccaceli Blasi, Maria Sole Borioni, Filippo Nuti, Daniele Belvisi, Marco Canevelli, Giovanni Fabbrini, Giuseppe Bruno

**Affiliations:** ^1^Department of Human Neuroscience, Sapienza University, Rome, Italy; ^2^Barcelonaβeta Brain Research Center (BBRC), Pasqual Maragall Foundation, Barcelona, Spain; ^3^Neurology Unit, IRCCS Neuromed, Pozzilli, Isernia, Italy; ^4^Aging Research Center, Department of Neurobiology, Care Sciences and Society, Karolinska Institutet and Stockholm University, Stockholm, Sweden

**Keywords:** autosomal dominant Alzheimer's disease, case report, medial temporal lobe hyperintensity, presenilin-1 mutation

## Abstract

**Introduction:**

Autosomal dominant Alzheimer's disease (ADAD), especially due to presenilin-1 (PSEN-1) gene mutations, may display a broad spectrum of clinical manifestations and neuroradiological findings. Occasionally, these manifestations may be rare and atypical, challenging the clinician's ability to recognize the disease. The description of the clinical characteristics and neuroradiological remarks of patients with specific mutations may improve clinicians' ability to identify them.

**Case Presentation:**

We report the case of a woman who presented with early-onset, rapidly progressive dementia associated with bilateral hyperintensity of the medial temporal lobe on T2-weighted MRI. After more common etiologies were excluded, genetic testing revealed a PSEN-1 C779T mutation. Notably, her brother, who carried the same mutation, did not exhibit these atypical neuroradiological findings.

**Conclusions:**

This case underscores the phenotypic variability associated with PSEN-1 mutations, even among individuals within the same family. Such variability and the possibility of atypical presentations may complicate the diagnostic process. In the presence of early-onset and rapidly progressive dementia associated with bilateral hyperintensity of the medial temporal lobe, ADAD and PSEN-1 mutation may be suspected and need to be addressed.

## 1. Introduction

Patients with autosomal dominant Alzheimer's disease (ADAD) may display highly variable clinical presentations and neuroradiological findings [1]. Occasionally, these manifestations may be rare and atypical, challenging the clinician's ability to recognize the disease and complicating differential diagnosis.

We report the case of a woman who presented with early-onset, rapidly progressive dementia associated with bilateral hyperintensity of the medial temporal lobe on T2-weighted magnetic resonance imaging (MRI). While signal changes in the medial temporal lobe on brain MRI are commonly associated with infectious or autoimmune encephalitis, such findings are infrequently linked to ADAD. After more common etiologies were excluded, genetic testing revealed a presenilin-1 (PSEN-1) C779T (Ala260Val) mutation. Notably, her brother, who carried the same mutation, did not exhibit these atypical neuroradiological findings.

This manuscript was prepared following the CARE (CAse REport) guidelines.

## 2. Case Presentation

A 43-year-old woman was admitted to our neurology ward with a six-month history of rapidly progressive memory and attention deficits, leading to difficulties in performing her job and daily living activities. She also exhibited depressive symptoms, persecutory delusions, and headaches. Her medical history included a multinodular thyroid goiter. Her family history was remarkable. Her mother died at 49 years in a road accident, with reports of early-onset cognitive decline of an undefined nature. Her father died by suicide at 43 years of age with a diagnosis of major depression. A maternal aunt who died at 40 years of age also presented with early-onset cognitive decline. Her 46-year-old brother resided in a long-term care facility due to early-onset dementia, but further clinical details were unavailable at the time. The family tree is included in the Supporting Information (Figure S1).

**Figure 1:**
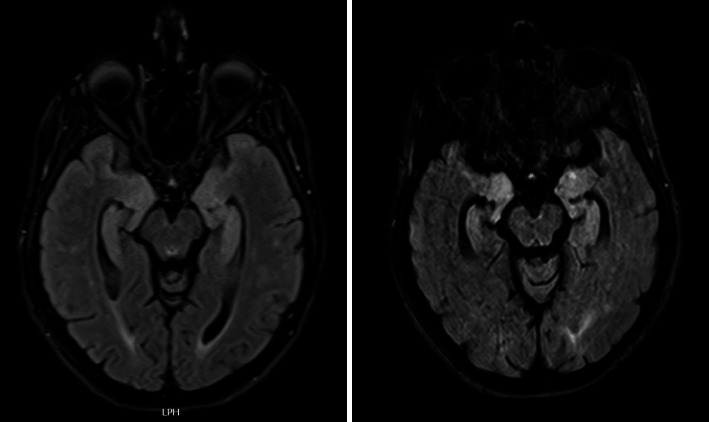
Fluid-attenuated inversion recovery (FLAIR) MRI sequences showing bilateral hyperintensity in the medial temporal lobes. The image on the left depicts the first MRI scan, while the image on the right illustrates the 6-month follow-up examination.

The neurological examination revealed spatial and temporal disorientation, attentional lability, ideomotor apraxia, and mild dysarthria. Brain MRI revealed bilateral hyperintensity in T2-weighted sequences involving the uncus, amygdalae, hippocampi, and parahippocampal gyrus (Figure 1). Viral or autoimmune encephalitis was suspected and ruled out by normal findings in cerebrospinal fluid, blood, and full-body computed tomography. Electroencephalography revealed widespread bursts of delta waves. A search for prion protein was performed on a nasal brush, and the result was negative. A more comprehensive cognitive assessment was attempted but not completed because of the patient's severe irritability and destructive behaviors. Finally, genetic tests (Supporting S2) identified a heterozygous C779T mutation in the PSEN-1 gene, classified as pathogenic according to ACMG guidelines (e.g., CADD-Phred 32), and confirmed by Sanger sequencing. Therefore, a diagnosis of ADAD was made. After the administration of typical and atypical neuroleptics, she developed severe parkinsonism. A follow-up MRI performed 6 months later showed persistent bilateral medial temporal lobes hyperintensity, together with a marked progression of atrophy (Figure 1).

**Figure 2:**
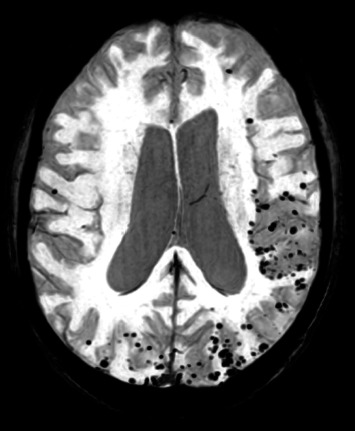
Gradient echo (GRE) MRI sequences demonstrating diffuse atrophy and multiple microbleeds.

Given the patient's ADAD diagnosis, a visit with her brother was scheduled. He had experienced cognitive decline since he was 39 years old. At the time of the visit, he was 46 years old and presented with severe, multidimensional cognitive impairment, visual hallucinations, and tonic–clonic seizures. Brain MRI revealed severe diffuse atrophy, chronic vascular encephalopathy, and several cerebral microbleeds (Figure 2). Genetic testing confirmed the presence of the same PSEN-1 C779T mutation, and a diagnosis of ADAD with cerebral amyloid angiopathy (CAA) was made.

## 3. Discussion

The PSEN-1 gene encodes PSEN-1, a multifunctional protein that plays an essential role in regulating membrane dynamics and protein trafficking and is involved in the catalytic function of the γ-secretase complex [2]. The PSEN-1 gene is located on the long arm of chromosome 14, and to date, more than 300 mutations have been described [3]. The C779T mutation leads to a missense substitution of alanine to valine in codon 260 of exon 8. This exon encodes the carboxy terminus of the 6 transmembrane hydrophobic domains [4], representing a slicing site and a possible hotspot for mutation. Most likely, it forms an important functional domain of presenilin proteins [5].

PSEN-1 mutation represents the most common cause of ADAD and is responsible for 43% of these cases [6]. It exhibits high penetrance transmission. The age of onset is variable, frequently early, and influenced by the specific mutation type [7]. Patients with these mutations usually develop an Alzheimer's disease clinical phenotype with early impairment of episodic memory and progressive involvement of multiple cognitive domains. Moreover, they tend to experience atypical cognitive symptoms with possible language and behavioral presentation (especially for exon 8 mutations) more frequently than sporadic cases do [8]. These patients may also present with myoclonus, seizures (with late or early onset), and pyramidal, extrapyramidal, and cerebellar signs [8]. In addition, atypical presentations with spastic paraparesis, frontotemporal degeneration, Parkinson's disease, Lewy body dementia, and cerebellar ataxia have been reported [1, 7]. Significant phenotypic heterogeneity has been observed, with great variability in individuals with the same mutation and in the same family, probably derived from other genetic and epigenetic factors [1] (i.e., APOE status [9]). Consequently, a specific genotype–phenotype relationship is unclear.

C779T mutations have been reported in three different case series involving families with Japanese, French, and English pedigrees [7, 10, 11]. These patients exhibited early onset (mean age 36.5 years, range 27–46 years), a mean age of death of 54 years (range 51–60), a mean duration of disease of 8 years, presentation with memory loss and personality changes, and, frequently, myoclonus and seizures at the late and end stages [7, 10, 11].

To date, no distinctive neuroradiological features have been consistently associated with the PSEN-1 C779T (Ala260Val) mutation. However, similar to other PSEN-1 mutations occurring beyond codon 200, CAA is frequently observed [12]. Recently, a case of this specific mutation has been reported with FLAIR-hyperintense foci in the white matter, suggesting that white matter changes may also be present [13]. Medial temporal lobe T2-weighted hyperintensities are frequently found in various diseases, such as herpes simplex encephalitis, neurosyphilis, limbic encephalitis, multiple sclerosis, mesial temporal sclerosis, and epilepsy [14]. Conversely, it is a rare finding in the context of ADAD, and to the best of our knowledge, it has been described only once in a different (e.g., S170F) PSEN-1 mutation [15]. This report represents the first description of medial temporal lobe hyperintensities associated with the PSEN-1 C779T mutation. However, the absence of this finding in the patient's affected sibling confirms the phenotypic variability and the difficulty in defining a specific genotype–phenotype association.

Several mechanisms and contributing factors may be hypothesized to explain this atypical MRI finding, including peri-ictal alterations occurring even in the absence of overt clinical seizures, inflammatory processes, or concomitant neuropathological conditions [16]. Furthermore, APOE genotyping has been shown to influence the clinical expression and, potentially, the radiological phenotype of PSEN-1-related Alzheimer's disease [9]. The limited ability to explore the full spectrum of potential causes underlying the medial temporal hyperintensity and to account for the radiological discrepancy between the two siblings represents a noteworthy limitation of this report. Therefore, the factors underlying the observed phenotypic variability remain to be elucidated, and future investigations combining genetic, neuroimaging, and longitudinal clinical data will be essential to characterize the clinical and radiological spectrum associated with PSEN-1 mutations.

## 4. Conclusion

Although such presentations are rare and atypical, in the presence of bilateral medial temporal lobe hyperintensities in patients with rapidly progressive, early-onset dementia, ADAD may be suspected. After more common and potentially reversible conditions are ruled out, genetic testing for ADAD should be performed on these patients.

## Data Availability

The data that support the findings of this study are available on request from the corresponding author. The data are not publicly available due to privacy or ethical restrictions.

## Nomenclature

ADAD: Autosomal dominant Alzheimer's disease
CAA: Cerebral amyloid angiopathy
MRI: Magnetic resonance imaging
PSEN-1: Presenilin-1

## Supporting Information

Figure S1. Family tree.

Supporting S2. List of the 63 genes included in the multigene dementia panel.
