# Supplementary material for: Presenilin-1 C779T Mutation Presenting With Rapidly Progressive Dementia and Medial Temporal Lobe MRI Changes
Source: Case Rep Neurol Med. 2025 Nov 14;2025:8251065. doi: 10.1155/crnm/8251065 (PMC12638162; doi:10.1155/crnm/8251065)
Supplement: Supporting Information — Additional supporting information can be found online in the Supporting Information section. [file 8251065.f1.docx]

**Figure S1**

Family tree

**
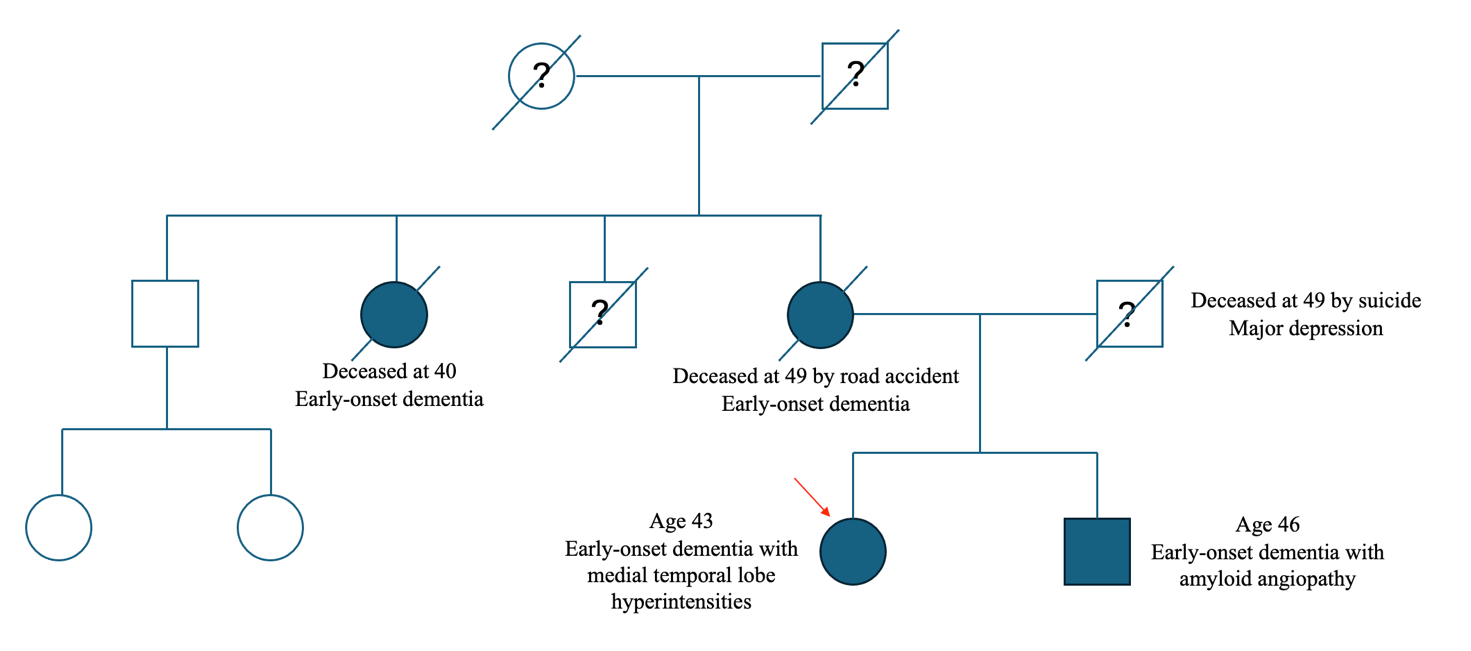
**

**Supporting S2**

List of the 63 genes included in the multigene dementia panel

AARS2 (alanyl-tRNA synthetase 2, mitochondrial), APP (amyloid beta precursor protein), ARSA (arylsulfatase A), ATP13A2 (ATPase cation transporting 13A2), ATP7B (ATPase copper transporting beta), CCNF (cyclin F), CHCHD10 (coiled-coil-helix-coiled-coil-helix domain containing 10), CHMP2B (charged multivesicular body protein 2B), CLN6 (CLN6 transmembrane ER protein), COL4A1 (collagen type IV alpha 1 chain), CP (ceruloplasmin), CSF1R (colony stimulating factor 1 receptor), CST3 (cystatin C), CTSF (cathepsin F), DCTN1 (dynactin subunit 1), DNAJC5 (DnaJ heat shock protein family member C5), EIF2B1 (eukaryotic translation initiation factor 2B subunit alpha), EIF2B2 (eukaryotic translation initiation factor 2B subunit beta), EIF2B3 (eukaryotic translation initiation factor 2B subunit gamma), EIF2B4 (eukaryotic translation initiation factor 2B subunit delta), EIF2B5 (eukaryotic translation initiation factor 2B subunit epsilon), FTL (ferritin light chain), FUS (FUS RNA binding protein), GALC (galactosylceramidase), GBA (glucosylceramidase beta), GFAP (glial fibrillary acidic protein), GLA (galactosidase alpha), GRN (granulin precursor), GSN (gelsolin), HEXA (hexosaminidase subunit alpha), HNRNPA2B1 (heterogeneous nuclear ribonucleoprotein A2/B1), HTRA1 (HtrA serine peptidase 1), ITM2B (integral membrane protein 2B), MAPT (microtubule associated protein tau), NOTCH3 (notch receptor 3), NPC1 (NPC intracellular cholesterol transporter 1), NPC2 (NPC intracellular cholesterol transporter 2), OPTN (optineurin), PANK2 (pantothenate kinase 2), PDGFB (platelet derived growth factor subunit B), PDGFRB (platelet derived growth factor receptor beta), PLA2G6 (phospholipase A2 group VI), PLP1 (proteolipid protein 1), PPT1 (palmitoyl-protein thioesterase 1), PRNP (prion protein), PSEN1 (presenilin 1), PSEN2 (presenilin 2), SERPINI1 (serpin family I member 1), SLC20A2 (solute carrier family 20 member 2), SQSTM1 (sequestosome 1), TARDBP (TAR DNA binding protein), TBK1 (TANK binding kinase 1), TIA1 (TIA1 cytotoxic granule associated RNA binding protein), TREM2 (triggering receptor expressed on myeloid cells 2), TREX1 (three prime repair exonuclease 1), TTR (transthyretin), TUBA4A (tubulin alpha 4A), TYROBP (transmembrane immune signaling adaptor TYROBP), UBQLN2 (ubiquilin 2), VCP (valosin containing protein), VPS13A (vacuolar protein sorting 13 homolog A), XK (X-linked Kx blood group), XPR1 (xenotropic and polytropic retrovirus receptor 1).
